# Current Perspectives and Unmet Needs of Primary Immunodeficiency Care in Asia Pacific

**DOI:** 10.3389/fimmu.2020.01605

**Published:** 2020-08-13

**Authors:** Daniel Leung, Gilbert T. Chua, Alric V. Mondragon, Youjia Zhong, Le Nguyen-Ngoc-Quynh, Kohsuke Imai, Pandiarajan Vignesh, Narissara Suratannon, Huawei Mao, Wen-I Lee, Yae-Jean Kim, Godfrey C. F. Chan, Woei Kang Liew, Le Thi Minh Huong, Hirokazu Kanegane, Dina Muktiarti, Xiaodong Zhao, Fatima Johanna Santos-Ocampo, Amir Hamzah Abdul Latiff, Reinhard Seger, Hans D. Ochs, Surjit Singh, Pamela P. Lee, Yu Lung Lau

**Affiliations:** ^1^Department of Pediatrics and Adolescent Medicine, Li Ka Shing Faculty of Medicine, The University of Hong Kong, Hong Kong, China; ^2^Department of Medicine, University of the Philippines—Philippine General Hospital, Manila, Philippines; ^3^Khoo Teck Puat-National University Children's Medical Institute, National University Health System, Singapore, Singapore; ^4^National Hospital of Pediatrics, Hanoi, Vietnam; ^5^Department of Community Pediatrics, Perinatal and Maternal Medicine, Tokyo Medical and Dental University, Tokyo, Japan; ^6^Department of Pediatrics, Advanced Pediatrics Centre, Postgraduate Institute of Medical Education and Research, Chandigarh, India; ^7^Pediatric Allergy and Clinical Immunology Research Unit, Division of Allergy and Immunology, Department of Pediatrics, Faculty of Medicine, Chulalongkorn University, King Chulalongkorn Memorial Hospital, Bangkok, Thailand; ^8^Children's Hospital of Chongqing Medical University, Chongqing, China; ^9^Primary Immunodeficiency Care and Research (PICAR) Institute, Chang Gung University College of Medicine, Chang Gung Memorial Hospital, Taoyuan, Taiwan; ^10^Department of Pediatrics, Samsung Medical Center, School of Medicine, Sungkyunkwan University, Seoul, South Korea; ^11^Rheumatology and Immunology Service, Department of Pediatric Medicine, KK Women's and Children's Hospital, Singapore, Singapore; ^12^Department of Child Health and Development, Tokyo Medical and Dental University, Tokyo, Japan; ^13^Department of Child Health, Faculty of Medicine, Universitas Indonesia – Cipto Mangunkusumo Hospital, Jakarta, Indonesia; ^14^Section of Allergy/Immunology, Department of Pediatrics, Makati Medical Center, Makati City, Philippines; ^15^Allergy & Immunology Centre, Pantai Hospital Kuala Lumpur, Kuala Lumpur, Malaysia; ^16^Division of Immunology/HSCT, University Children's Hospital Zürich, Zürich, Switzerland; ^17^Department of Pediatrics, Seattle Children's Research Institute, University of Washington, Seattle, WA, United States

**Keywords:** primary immunodeficiencies, resource needs, health resources, immunology, standard of care, specialty training

## Abstract

**Background:** The Asia Pacific Society for Immunodeficiencies (APSID) conducted nine primary immunodeficiency (PID) Schools in 5 years since inauguration to provide PID care training for early career physicians in Asia Pacific, a region with divergent needs in PID resources and training.

**Objective:** To identify differences in PID patient care resource and training needs across Asia Pacific and propose a corresponding action plan.

**Methods:** The Human Development Index (HDI) indicates the degree of socio-economic development in each country/region. Information related to investigations and learning issues were extracted from the abstracts and personal statements from all Schools and mapped onto resource and training needs. Correlations between HDI and country/region-specific parameters were tested by two-tailed Pearson correlation.

**Results:** A total of 427 abstracts were received in nine Schools between 2015 and 2020, predominantly on immunodeficiencies affecting cellular and humoral immunity. Genetic confirmation was described in 61.8% of abstracts, and its absence negatively correlated with HDI (*r* = −0.696, *p* = 0.004). Essential immunologic and genetic tests were not available in 25.4 and 29.5% of abstracts, respectively, and their absence negatively correlated with HDI (*r* = −0.788, *p* < 0.001; *r* = −0.739, *p* = 0.002). HDI positively correlated with average testing level (*r* = 0.742, *p* = 0.002). Cases from medium-HDI countries/regions focused on learning how to investigate a patient for PIDs in cases of severe or atypical infections, whereas those from very-high-HDI countries/regions, from which most faculty members originated, listed hematopoietic stem cell transplantation and gene therapy, newborn screening, and research as learning issues more frequently.

**Conclusion:** There are unique HDI-related PID resource and training needs in each country/region. APSID proposes HDI group-specific strategies to improve PID care and education in her member countries/regions. Further quantitative analysis of needs in PID care in Asia Pacific is needed for lobbying governments to increase their support for PID care and research.

## Introduction

Primary immunodeficiencies (PIDs), also known as inborn errors of immunity (IEIs), consist of more than 400 monogenic diseases. Individually, most PIDs are rare, yet together have a prevalence of about 1 in 1000–5000 (1). Increased access to next-generation sequencing (NGS) has enabled the discovery of novel PID genes and phenotypes resulting in more patients diagnosed with PIDs in recent years. Despite advances in the diagnosis and treatment of PIDs, resource-limited countries may not be equipped with the diagnostic and therapeutic options necessary for the management of PIDs (2).

The Asia Pacific region encompasses more than 45 countries with 4.6 billion inhabitants, accounting for 60% of the global population (3). There exist large differences in various facets of human development such as health, standard of living, and education across Asia Pacific, as reflected by the United Nations Development Program 2019 Human Development Index (HDI), which result in significant differences in the level of care available for patients with rare diseases in each country/region (4). Countries/regions of very high HDIs such as Hong Kong and Japan have adequate expertise and resources for the diagnosis and management of PIDs along with structured pediatric and adult immunology training programs, whereas these are lacking in countries with medium HDIs such as Myanmar and Cambodia (5).

To promote the development of PID care and training in the Asia Pacific region, the Asia Pacific Society for Immunodeficiencies (APSID) was conceptualized in Osaka in 2015 and inaugurated in Hong Kong in 2016. APSID has regularly organized PID Schools (“Schools”) between 2015 and 2020 for early-career physicians and scientists (“students”) from Asia Pacific. Some of the Schools were organized alongside with national or international conferences organized by immunologic or pediatric societies to maximize the reach of the Schools in the medical community. Similar educational events have been organized by PID societies in other continents and across other specialties (6, 7).

So far, analyses delineating the spectrum of PID care and training needs in Asia Pacific have yet to be performed. In this study, we aimed to analyze the descriptive information from the APSID School abstracts to identify the resource and training needs of PID care in settings with different HDI, in order to propose an action plan to further enhance PID care and education in the region.

## Methods and Materials

### APSID Schools

The framework of APSID Schools is based on the experience of the postgraduate Pediatric Immunology and Infectious Diseases subspecialty study day in Hong Kong, which was effective in improving trainees' knowledge related to PIDs (8). As part of the application to attend the Schools, students were invited to submit abstracts of any suspected or confirmed PID cases, along with the learning objectives highlighting the clinical and practical challenges encountered, and personal statements narrating their aspirations to develop PID service and research in their countries/regions. Each School was a 1- or 2-day event, with each session consisting of an interactive seminar conducted by an expert on a particular PID topic, e.g., combined immunodeficiencies, followed by three or four short case presentations related to that topic. Each case presentation was closed by an open-floor discussion with faculty members, students, and observers sharing their experiences in managing these patients. The faculty members would provide oral and written feedback to the students. At the end of each School, students filled out an evaluation form for the organizers, and, beginning from the 2019 Manado School, participated in an online quiz on topics discussed using their mobile phones.

### Data Collection

Abstracts submitted to all APSID Schools held between 2015 and 2020 were analyzed. Each abstract provided the student's name, affiliation and country of origin, a title, a 400-word main text, up to three key learning points, and a personal statement. Students were informed by email of the study, and that their names were blinded for the analysis, retaining information on the countries/regions and centers only.

### Study Design

Investigators first familiarized themselves with the content of the abstracts and extracted themes to form the initial coding and analysis strategies, according to which an initial analysis of all abstracts was performed. The final coding and analysis strategies, as detailed below, were revised based on the initial analysis. Coding was repeated twice to ensure accuracy.

### Diagnoses

The diagnoses were grouped according to the 2019 update of the classification by the International Union of Immunological Societies (IUIS) Expert Committee on IEI based on clinical phenotypes and molecular etiology (1). Cases without genetic diagnoses were labeled as genetically undiagnosed, including cases for which genetic testing was not performed, or with inconclusive genetic test results.

### Resource Needs

To investigate the resource needs, we analyzed how sophisticated a given case was investigated with immunologic and genetic testing. Each abstract was categorized into one of six levels of immunologic testing (0–5) (Table 1). The testing levels were based on the modified Jeffrey Modell Foundation Four Stages of Testing for Immunodeficiencies (9). Level 0 represents that no PID-related immunologic tests were mentioned for the case, whereas level 5 represents that highly sophisticated tests were performed, such as tests of protein expression or function. Details of how testing levels were coded are described in Table 1. The mean testing level achieved by a country/region was calculated by adding up the test levels of abstracts presented by that country/region divided by the number of abstracts presented by that country/region. The mean testing level was taken as an estimate of the depth of investigation of cases suspected as PIDs from each country/region.

**Table 1:** Coding of testing levels: testing levels are assigned by the highest level of test achieved according to this table (0–5).

**Level**	**Presence of any one of these tests**
0	No laboratory tests performed
1	CBC, IgG/A/M
2	IgE, C3, C4, CH50, AH50, functional antibody response including to tetanus, polio, hepatitis B vaccine
3	Basic lymphocyte subset analysis (CD3, CD4, CD8, CD19, CD20, CD16, CD56), dihydrorhodamine test, nitroblue tetrazolium test
4	Lymphocyte cell marker CD45RO/RA, TREC qPCR, lymphocyte proliferation test, NK cytotoxicity studies
5	Comprehensive T or B cell subset with markers other than those mentioned in levels 3 and 4, protein expression of PID genes (e.g., WASP, BTK, CD40L by flow cytometry), assays of pathway activation (e.g., pSTAT3 by Western blot or flow cytometry), cytokine production (e.g., IFN-gamma level by ELISA)

*CBC, complete blood count, TREC, T-cell receptor excision circle; qPCR, quantitative polymerase chain reaction*.

Resource needs were categorized into “unavailable essential immunologic testing” and “unavailable genetic testing.” Unavailable essential immunologic testing implied the lack of access to PID diagnostic resources, or the level of immunologic testing was coded below level 3, or stated level 3 immunologic testing was incomprehensive; e.g., flow cytometry-based lymphocyte immunophenotyping was limited to CD3 and CD19/20 subsets without CD16/56 for SCID. Abstracts were categorized as “unavailable genetic testing” when genetic testing was not mentioned, or a non-local institution was stated to have performed the test.

### Training Needs

To investigate the training needs, learning issues were organized into three categories: “diagnosis, general investigations and microbiology,” “hematopoietic stem cell transplant (HSCT) and gene therapy, newborn screening (NBS) and research,” and “others.” “Others" includes epidemiology, treatment, genetic testing, pathophysiology, counseling issues, prognosis, and other topics. General investigations represented tests other than genetic testing, e.g., X-ray, complete blood count (CBC). To look into the availability of educators within Asia Pacific, faculty members and their country/region of origin were also analyzed.

### Exclusion Criteria

Abstracts without sufficient details about investigations conducted, e.g., reports limited to HSCT outcomes; or abstracts not referring to, or not suspecting a diagnosis of PIDs, e.g., secondary hemophagocytic lymphohistiocytosis or pediatric idiopathic systemic lupus erythematosus, were excluded from the coding and analysis of resource needs. Cases from countries/regions outside of Asia Pacific, e.g., USA, are not included for country/region-specific analyses only.

### Statistical Analysis

Data entry and statistical analysis were performed using IBM SPSS Statistics version 25 (Armonk, New York, USA) and Microsoft Excel® (Redmond, Washington, USA). Mean values with or without standard deviation were obtained for country/region-specific parameters. Correlations between HDI and country/region-specific parameters were tested by two-tailed Pearson correlation.

### Results Interpretation and Action Plan Proposal

Interpretation and discussion of the results, as well as an action plan addressing the needs were proposed by the primary authors, then critically reviewed and further contributed by co-authors of this manuscript, who are clinicians or academics interested in PID, affiliated to institutions and PID societies in different parts of Asia Pacific, Europe, and America.

## Results

### Participants Demographic Profile

**Figure 1:**
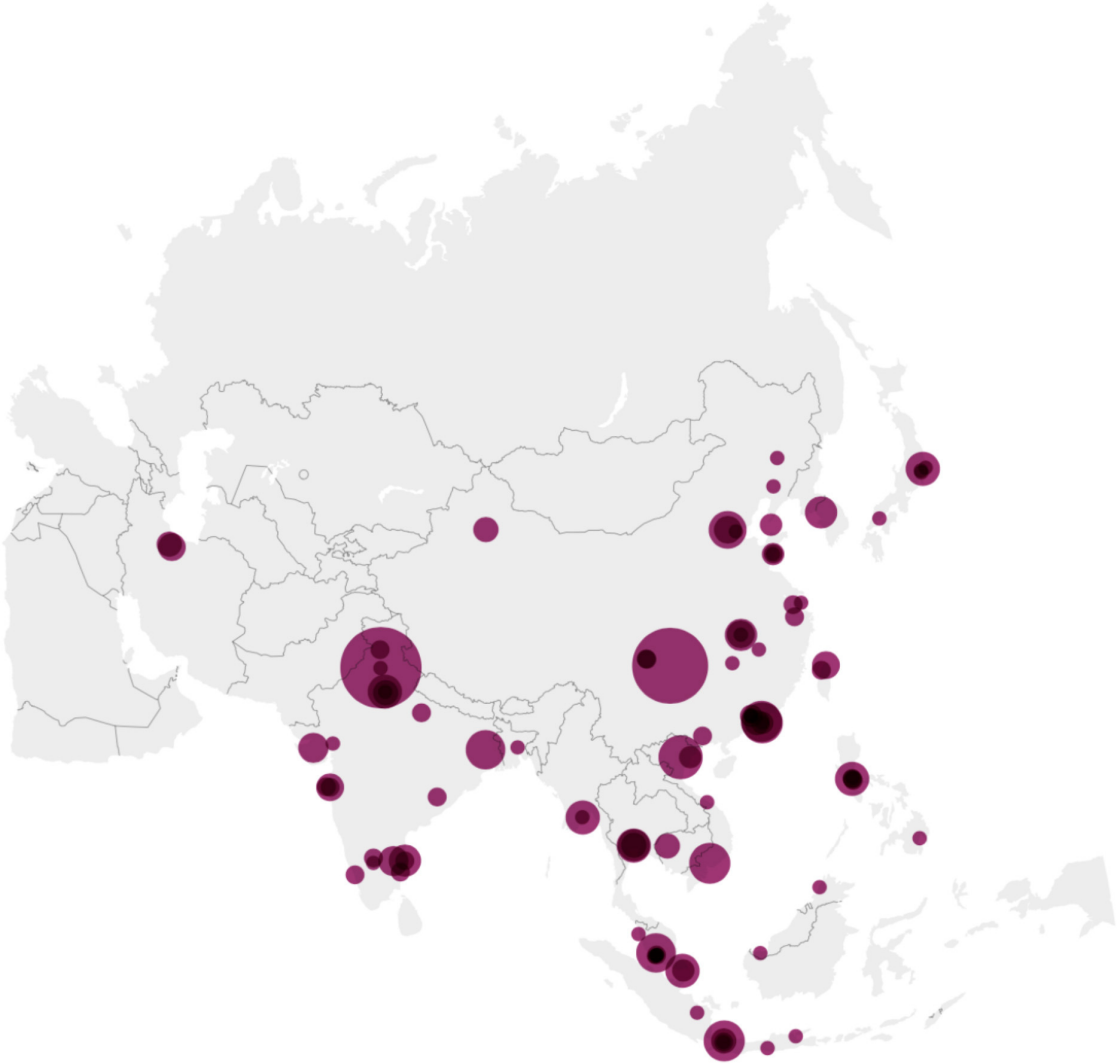
Origin of abstracts on the map of Asia. There were 423 abstracts submitted to nine APSID Schools from 99 centers in Asia Pacific between 2015 and 2020, ranging from 1 to 52 per center. The larger the circle, the higher the number of abstracts. Created with Datawrapper.

A total of 427 abstracts presented by students from 103 centers in 19 countries/regions in 9 APSID Schools conducted between 2015 and 2020 were analyzed (Figure 1). The countries/regions with the highest number of abstracts presented were mainland China and India, contributing 115 and 107 abstracts, respectively (Supplementary Table 1). Countries/regions with medium (HDI 0.550–0.699), high (HDI 0.700–0.799), and very high degree of human development (HDI 0.800–1) accounted for 164, 161, and 102 abstracts, respectively.

### Diagnoses

**Figure 2:**
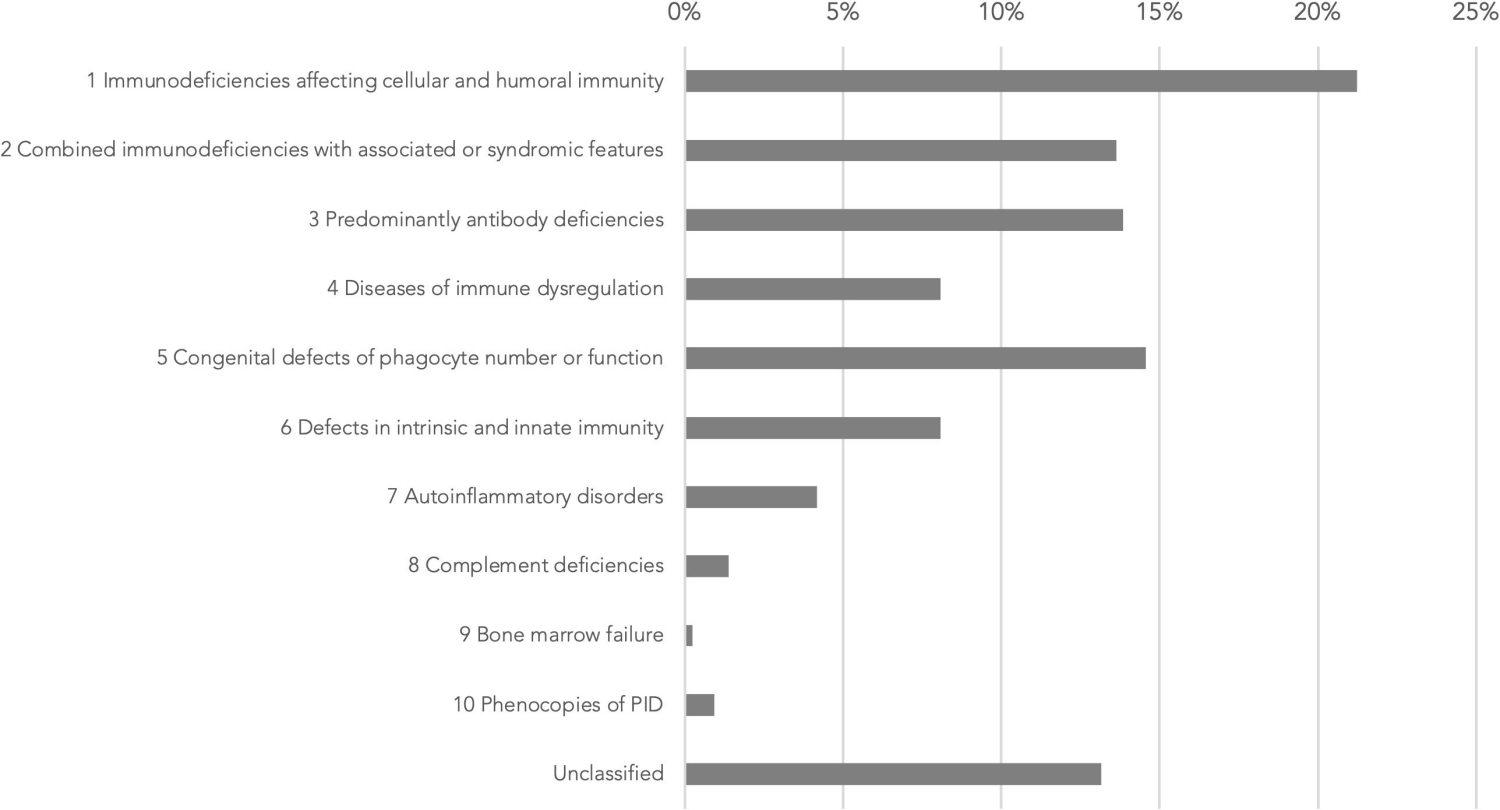
Genetic diagnoses in IUIS IEI classification 2019 update (1). IUIS: International Union of Immunological Societies, IEI: inborn errors of immunity, PID: primary immunodeficiency.

Based on the 2019 update of the IEI classification by the IUIS Expert Committee, the most commonly presented cases belonged to immunodeficiencies affecting cellular and humoral immunity (21.4%), congenital defects of phagocyte number or function (14.7%), predominantly antibody deficiencies (14.0%), and combined immunodeficiencies with associated or syndromic features (13.7%) (Figure 2). Some were considered as unclassified as they are inborn errors of metabolism, e.g., *SGPL1* deficiency, or chromosomal disorders, e.g., trisomy 21.

### Resource Needs

#### Investigations

**Figure 3:**
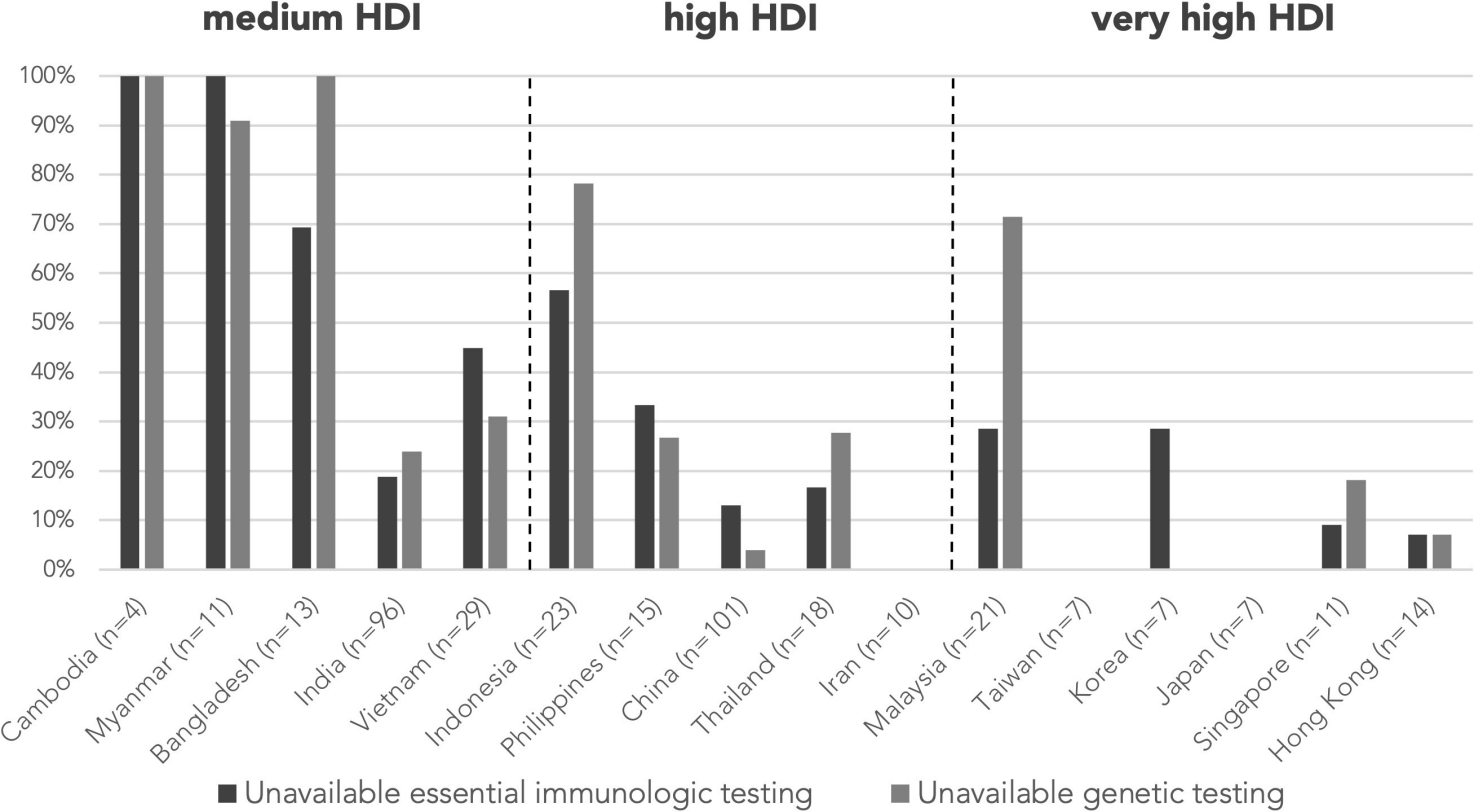
Proportion of cases without essential immunologic testing and genetic testing by country/region. Essential immunologic tests are tests classified level 3–5 according to Table 1. The proportion of cases without essential immunologic and genetic testing negatively correlated with HDI respectively by two-tailed Pearson correlation (*r* = −0.788, *p* < 0.001; *r* = −0.739, *p* = 0.002). *n* is the number of abstracts from that country/region included in this analysis. Countries/regions are in ascending order according to their HDI from the left. HDI, Human Development Index.

**Figure 4:**
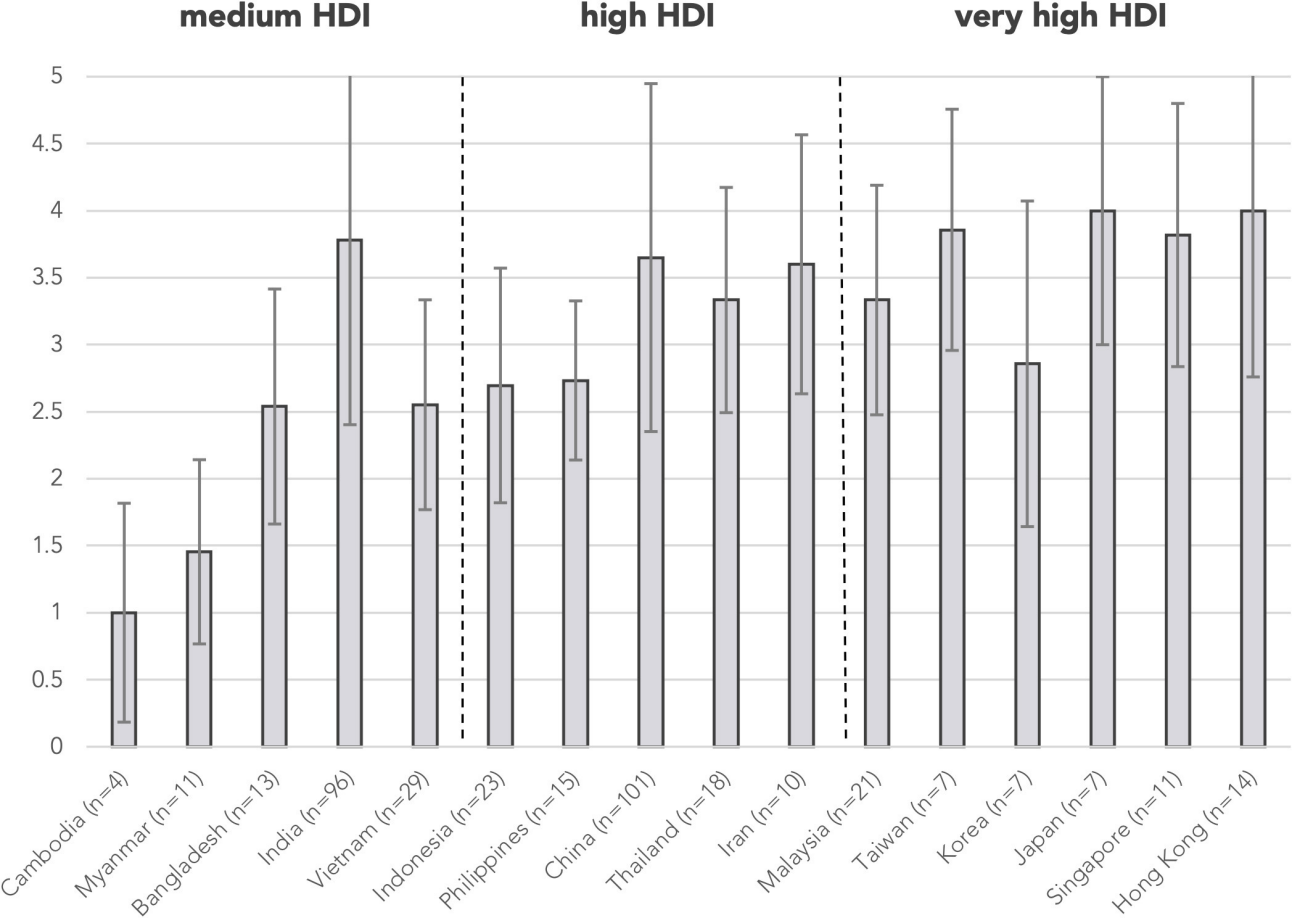
Mean testing level by country/region. Testing levels are assigned according to Table 1 for each abstract. Mean testing level positively correlated with HDI by two-tailed Pearson correlation (*r* = 0.742, *p* = 0.002). Error bars show the range of ±1 S.D. of mean testing level of cases from the country/region. *n* is the number of abstracts from that country/region included in this analysis. Countries/regions are in ascending order according to their HDI from the left. HDI, Human Development Index.

Testing levels were not assigned for 38 abstracts as the abstracts did not focus on investigations, e.g., reports describing only HSCT in patients with PID, and are excluded from the analysis of resource needs. A total of 99 abstracts (25.4%) were classified as “unavailable essential immunologic testing” by not describing tests at or above level 3. The proportion of abstracts from countries/regions with unavailable essential immunologic testing are illustrated in Figure 3. The majority of these abstracts were clustered in the countries/regions of medium or high HDIs, including Cambodia (100%), Bangladesh (69.2%), Myanmar (100%), Vietnam (44.8%), and Indonesia (56.5%). The proportion of cases without essential immunologic testing negatively correlated with HDI respectively by two-tailed Pearson correlation (*r* = −0.788, *p* < 0.001). The average testing levels achieved in abstracts also positively correlated with HDI (*r* = 0.742, *p* = 0.002). Cambodia and Myanmar did not reach the average testing level of 2 (Figure 4).

#### Genetic Testing

**Figure 5:**
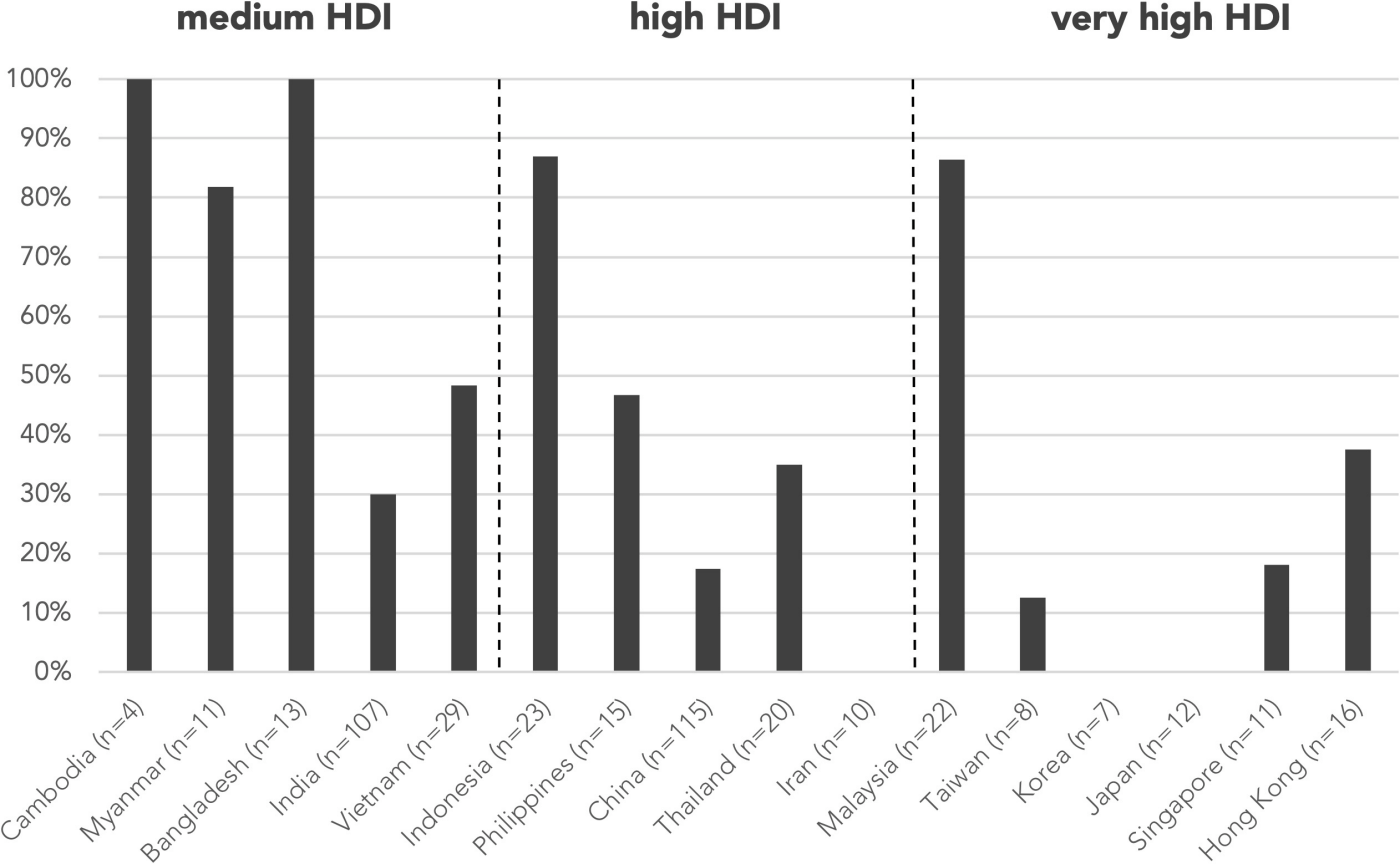
Proportion of genetically undiagnosed cases by country/region. Proportion of genetically undiagnosed cases negatively correlated with HDI by two-tailed Pearson correlation (*r* = −0.696, *p* = 0.004). *n* is the number of abstracts from that country/region included in this analysis. Countries/regions are in ascending order according to their HDI from the left. HDI, Human Development Index.

A genetic diagnosis was provided in 264 (61.8%) abstracts, while the remaining 163 (38.2%) were classified as genetically undiagnosed, with country/region-specific data in Figure 5. Proportion of genetically undiagnosed cases negatively correlated with HDI by two-tailed Pearson correlation (*r* = −0.696, *p* = 0.004). Among the 389 abstracts included in analysis of resource needs, genetic testing was not mentioned/performed or was performed outside of home country/region (“unavailable genetic testing”) in 109 abstracts (28.0%) and 6 abstracts (1.5%), respectively. The countries/regions that assisted with the genetic tests included Hong Kong, Italy, Japan, and the USA, all with very high HDI. The majority of the abstracts with no genetic testing or with testing outside home country/region were from medium-HDI countries/regions, including Cambodia (100%), Myanmar (91.0%), and Bangladesh (100%), except Indonesia (78.3%), which belongs to the high-HDI category (Figure 3). The proportion of cases without genetic testing negatively correlated with HDI respectively by two-tailed Pearson correlation (*r* = −0.739, *p* = 0.002). Genetic testing results were negative or pending in 41 abstracts (10.5%).

### Training Needs

#### Learning Issues

**Figure 6:**
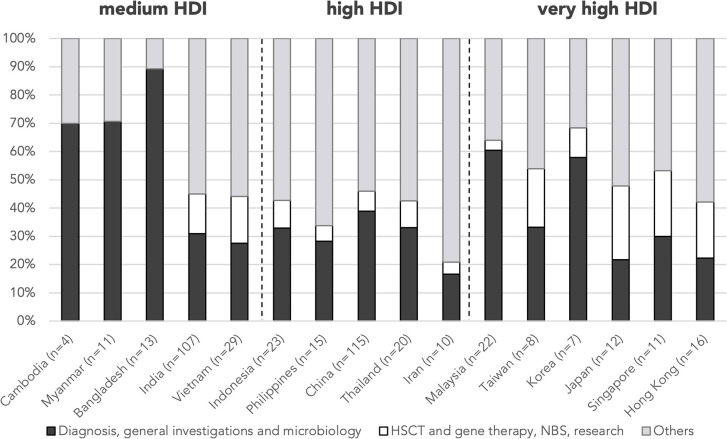
Proportion of learning issues by country/region. *n* is the number of abstracts from that country/region included in this analysis. Countries/regions are in ascending order according to their HDI from the left. HDI, Human Development Index; HSCT, hematopoietic stem cell transplant; NBS, newborn screening.

As to country/region-specific learning objectives, students from medium-HDI countries/regions focused predominantly on the diagnosis, general investigations, and microbiology (Cambodia 70.0%, Myanmar 70.6%, and Bangladesh 89.3%), whereas abstracts from very-high-HDI countries/regions listed more frequently HSCT/gene therapy, NBS, and research as learning issues (Japan 21.7%, Singapore 19.9%, Hong Kong 19.8%, and Taiwan 16.6%) compared with the mean (9.5%) (Figure 6). Vietnamese students, despite belonging to a medium-HDI country, also listed HSCT/gene therapy as a learning issue more frequently than the mean (16.6%).

#### Origin of Faculty

The nine APSID Schools featured a total of 46 faculty members from institutions in 12 Asian (26 faculty members) and 8 non-Asian (20 faculty members) countries/regions. The majority of the faculty members were from high- and very-high-HDI countries/regions (Supplementary Table 2).

## Discussion

The analysis of the School abstracts revealed the different needs in PID care and training across different HDI categories in the Asia Pacific region. Abstracts presented from the very-high-HDI countries/regions such as Hong Kong, Singapore, Japan, and Taiwan generally described more in-depth investigations and management. Students from these countries/regions prioritize their learning objectives in more advanced topics such as HSCT, NBS, and research. Students from Cambodia, Bangladesh, Myanmar, and Indonesia, with medium or high HDI, generally reported a lack in PID diagnostic tools. India and mainland China have an extremely large population size, allowing the pooling of human, laboratory, and other clinical resources in national specialized centers to support research and clinical care of rare diseases, resulting in better PID care despite belonging to the medium- and high-HDI categories respectively.

Making an accurate diagnosis is the cornerstone of managing rare diseases, yet it could be particularly challenging in medium-HDI countries/regions. This is well reflected in the analysis of learning issues. In Cambodia and Myanmar, where PID training is in the early phase of development, students reported difficulties in identifying and categorizing PIDs, and therefore prioritized learning to look for diagnostic clues related to specific PIDs in patients with severe infections (10). The Children's Hospital of Zürich and the Swiss Foundation of the Kantha Bopha Hospitals in Cambodia are collaborating to pilot a pediatric immunology and infectious disease training program, to be delivered by experienced local and overseas tutors with online exchanges. Other countries/regions with medium/high HDI such as Indonesia and the Philippines may not be able to provide technical and financial support for immunologic tests, similar to some nations in Latin America, the Middle East, or Africa, and instead may have to rely on phenotypic criteria or clinical scoring systems (11–15). Commercial availability of NGS services and the rapid decline in cost have allowed genetic diagnosis for rare diseases to be more accessible and affordable (16). Genotyping microarrays targeting known pathogenic variants in PID can be a low-cost strategy to facilitate genetic screening in areas with no access to traditional methods of genetic diagnosis (17). Alternatively, international PID molecular diagnostic and research networks led by quaternary centers in very-high-HDI countries/regions like the Asian Primary Immunodeficiency Network (APID Network) established by the University of Hong Kong have been offering free sequencing and e-consultation to patients in countries/regions where advanced immunologic testing or clinical genetics services are deficient (18, 19). Specimens such as extracted DNA, EDTA blood tubes, and dried blood spots can be couriered to overseas centers for sequencing and advanced immunologic testing (20, 21). Universal NBS for PIDs such as SCID, noted to be a learning priority by students from very-high-HDI countries/regions, has yet to be adopted by most healthcare systems in Asia Pacific. That is despite the fact that many countries/regions have acquired adequate experience in HSCT for PID, and many countries outside Asia have enjoyed success with NBS for SCID using T-cell receptor excision circle (TREC) (22–25).

Treatment availability is limited by healthcare budget and access to technology in Asia Pacific. Although antimicrobial prophylaxis, such as co-trimoxazole, is available in many countries/regions, the decision to start treatment may often be empirical when achieving an accurate diagnosis is difficult. Biologics, intravenous and subcutaneous immunoglobulin (IVIG/SCIG), and HSCT are unavailable or inaccessible to financially deprived patients in many medium- and high-HDI countries/regions. The Polish experience showed that participation in European Society for Immunodeficiencies (ESID) along with the combined efforts of physicians, patients, and government could lead to increased access of therapeutic options and improved quality of life of PID patients (26). In our study, Vietnamese students, despite belonging to a medium-HDI country, listed HSCT/gene therapy as a learning issue more frequently. This is likely explicable by the recent rapid development of HSCT in Vietnam, which was reported to have the largest increase in the number of HSCT performed in Asia Pacific in the past decade (27). HSCT is also mentioned as a learning issue by most other countries/regions in high- and very-high-HDI groups, and by India, also in the medium-HDI group, in line with the general increase of HSCT in Asia Pacific (28, 29). However, in countries/regions across all HDI groups in Asia, gene therapy remains unavailable as research and development of novel therapeutic options generally take place in North America and Europe.

Despite the availability of PID experts in Asia Pacific, training needs remain to be a significant issue. Structured PID training programs are commonly unavailable in medium- and high-HDI countries/regions and some very-high-HDI countries/regions (30). Most faculty members of APSID Schools originated from very-high-HDI countries/regions in or out of Asia. Medical schools in Asia Pacific may not cover PID in undergraduate curricula. Many students who attended the APSID Schools identified themselves as subspecialists in pediatric allergy, infectious disease, hematology, intensive care, respirology, gastroenterology, neurology, etc., as the primary clinicians managing PID patients in Asia, especially in countries without structured pediatric immunology training programs.

To address these training and resource needs, we suggest the following 5-year action plan for APSID. APSID will continue hosting regular Schools free of charge. Funding for trainees, especially those from medium-HDI countries/regions, must be provided to reduce the existing financial barriers. Supporting young pediatrics trainees to well-established PID centers overseas may be necessary to initiate the development of pediatric immunology in medium-HDI countries/regions. APSID should help national society members lobby governments to recognize pediatric immunology as a subspecialty and fund training programs. Investment in a national center for PID care and research will quicken the development of PID care in the country, taking the Postgraduate Institute of Medical Education and Research, Chandigarh and the National Institute of Immunohematology, Mumbai in India as examples (31, 32). National or regional centers devoted to PIDs should be equipped with diagnostic and therapeutic resources of a standard corresponding to the HDI, which we propose in Table 2. In the UK, PID care and research are spearheaded by two supraregional PID transplantation centers: Great North Children's Hospital in Newcastle upon Tyne and Great Ormond Street Hospital in London, each serving a population of around 35 million, together with 36 other recognized immunology centers (33). In Asia Pacific, governments should be encouraged to establish one national or regional PID referral center per 50–100 million citizens, with each center obligated to form local referral networks with every children's hospital in its catchment area. Alternatively, in Japan, the Primary Immunodeficiency Database in Japan (PIDJ) network project started in 2008 for clinicians in local hospitals to consult PID specialists in regional universities on the diagnosis and management of suspected PID cases, with the support of a centralized platform for immunologic and genetic analyses (34). The choice of which of these two models to follow depends on factors including the size of the population and the country, as well as how the healthcare system is organized. APSID will also keep a regularly updated list of international referral centers for diagnostic resources or HSCT in Asia Pacific on her website (paed.hku.hk/apsid) for physicians and patients. Enhancing the awareness of PID by doctors who are not specialized in these disorders may be tackled by creating internet-based resources in different languages. Establishing PID patient support groups and strengthening existing ones could raise awareness and promote advocacy. An APSID PID patient registry, modeled after the registry built by ESID or the Latin American Society for Immunodeficiencies (LASID), is essential to provide epidemiological data supporting the negotiations with local governments to fund clinical services, education, and research (35, 36). Existing regional and national PID networks with professional and patient societies, such as Asia Pacific Association of Allergy, Asthma and Clinical Immunology (APAAACI) and South East Asia PID (SEAPID), must come together for the betterment of lives of PID patients in the Asia Pacific region.

**Table 2:** The Asia Pacific Society for Immunodeficiencies (APSID) PID care resource standard for national/regional healthcare systems in different HDI groups in the Asia Pacific region.

**HDI**	**Diagnostics**	**Therapeutics**
Medium	1. CBC 2. IgG/A/M/E 3. Lymphocyte subset (CD3, CD4, CD8, CD19/20, and CD16/56) 4. NBT test or DHR test 5. C3, C4, CH50, and AH50 6. Functional antibody response to tetanus, polio, and hepatitis B vaccine 7. Sanger sequencing for *BTK, WAS, IL2RG*, and *CYBB*	1. Antimicrobials 2. IVIG
High	1. Naive and memory T cell marker CD45RO/RA 2. TREC qPCR 3. Lymphocyte proliferation test 4. NK cytotoxicity study 5. C1 esterase inhibitor level and function 6. Targeted PID NGS panel 7. WES	1. Commonly used biologics 2. HSCT 3. SCIG
Very high	1. Comprehensive T or B cell subset with markers other than those mentioned (CD3, CD4, CD8, CD19/20, CD16/56, CD45RO/RA) 2. Protein expression of PID genes (e.g., WASP, BTK, CD40L by flow cytometry) 3. Assays of pathway activation (e.g., pSTAT3 by Western blot or flow cytometry) 4. Cytokine production (e.g., IFN-gamma level by ELISA) 5. SCID NBS (TREC qPCR) 6. WGS	1. Complete access to biologics 2. Gene therapy

*HDI, Human Development Index (4); CBC, complete blood count; IVIG, intravenous immunoglobulin; NBT, nitroblue tetrazolium; DHR, dihydrorhodamine; TREC, T-cell receptor excision circle; qPCR, quantitative polymerase chain reaction; HSCT, hematopoietic stem cell transplantation; SCIG, subcutaneous immunoglobulin; PID, primary immunodeficiency; NGS, next-generation sequencing; WES, whole exome sequencing; SCID, severe combined immunodeficiency; NBS, newborn screening; WGS, whole genome sequencing*.

## Limitations

This study was limited by not being a quantitative epidemiological study and therefore data collected related to the diagnoses and availability of PID care and training only showed the approximate but not the exact situation. Some kinds of cases might have been overrepresented or underrepresented in the Schools as they were submitted based on the students' interest and the difficulty of the case. To avoid overinterpretation and misinterpretation, conclusions were only drawn when the data clearly suggested such phenomena and they were supported by external sources. Discussions were also contributed and checked by authors involved in PID care and development in different parts of Asia Pacific. With respect to availability of PID care resources, treatment options such as access to HSCT, immunoglobulin replacement therapy, or biologics were not analyzed as they were rarely mentioned in students' abstracts. The assignment of testing level was not perfect as there were PIDs where listed “essential” immunologic investigations are not vital to diagnosis; e.g., for severe congenital neutropenia, only CBC and bone marrow aspirate are required to make the phenotypic diagnosis. It might also overestimate the testing level received by all PID patients in a country/region as missed PID cases could not be presented and included.

## Conclusion

The analysis of a large number of APSID School abstracts provides a look through the keyhole into the current situation of PID care in the Asia Pacific region. Different countries/regions in Asia Pacific are at different stages of development in the care and training related to PID, depending on their HDI. APSID will continue to serve as an important platform for the sharing of experiences and resources to improve PID care in the Asia Pacific region.

## Data Availability Statement

The raw data supporting the conclusions of this article will be made available by the authors, without undue reservation.

## Author Contributions

YL, PL, HO, RS, HK, KI, SS, WL, XZ, GCFC, AM, LH, AL, PV, HM, NS, DM, FS-O, Y-JK, and W-IL organized APSID Schools. KI, YL, PL, GTC, AM, YZ, and LN-N-Q conceptualized the study. DL, GTC, AM, YZ, and LN-N-Q planned the study. DL and GTC carried out data entry and analysis, under supervision by YL and PL. DL, GTC, PL, and YL wrote the manuscript, which is critically revised by HO, RS, HK, KI, SS, WL, XZ, GCFC, AM, YZ, LN-N-Q, LH, AL, PV, HM, NS, DM, FS, YK, and W-IL. All authors contributed to the article and approved the submitted version.

## Conflict of Interest

The authors declare that the research was conducted in the absence of any commercial or financial relationships that could be construed as a potential conflict of interest.

## Abbreviations

APAAACI: Asia Pacific Association of Allergy, Asthma and Clinical Immunology
APID Network: Asian Primary Immunodeficiency Network
APSID: Asia Pacific Society for Immunodeficiencies
CBC: complete blood count
DHR: dihydrorhodamine
ESID: European Society for Immunodeficiencies
HDI: Human Development Index
HSCT: hematopoietic stem cell transplantation
IEIs: inborn errors of immunity
IUIS: International Union of Immunological Societies
IVIG: intravenous immunoglobulin
LASID: Latin American Society for Immunodeficiencies
NBS: newborn screening
NBT: nitroblue tetrazolium
NGS: next-generation sequencing
PIDs: primary immunodeficiencies
qPCR: quantitative polymerase chain reaction
SCID: severe combined immunodeficiency
SCIG: subcutaneous immunoglobulin
SEAPID: South East Asia PID
TREC: T-cell receptor excision circles
WES: whole exome sequencing
WGS: whole genome sequencing.
