# Supplementary material for: Current Perspectives and Unmet Needs of Primary Immunodeficiency Care in Asia Pacific
Source: Front Immunol. 2020 Aug 13;11:1605. doi: 10.3389/fimmu.2020.01605 (PMC7438539; doi:10.3389/fimmu.2020.01605)
Supplement: Supplementary file 1 [file Data_Sheet_1.pdf]

Supplementary Materials

Current Perspectives and Unmet Needs of Primary Immunodeficiency Care in Asia Pacific

Daniel Leung*, Gilbert T. Chua*, Alric V. Mondragon, Youjia Zhong, Le Nguyen-Ngoc-Quynh, Kohsuke Imai, Pandiarajan Vignesh, Narissara Suratannon, Huawei Mao, Wen-I Lee, Yae-Jean Kim, Godfrey C.F. Chan, Woei Kang Liew, Le Thi Minh Huong, Hirokazu Kanegane, Dina Muktiarti, Xiaodong Zhao, Fatima Johanna Santos-Ocampo, Amir Hamzah Abdul Latiff, Reinhard Seger, Hans D. Ochs, Surjit Singh, Pamela P. Lee[+] and Yu Lung Lau[+]

* Co-first authors, + Co-corresponding authors

Corresponding Authors

Yu Lung Lau (email: lauylung@hku.hk), Pamela P. Lee (email: ppwlee@hku.hk)

Address: 1/F, New Clinical Building, Department of Pediatrics and Adolescent Medicine, Queen Mary Hospital, 102 Pokfulam Road, Hong Kong.

Tel: (852) 2255-4482; Fax: (852) 2255-4089

Supplementary Table 1. Number of abstracts by country/region. Taiwan is not given an HDI 2019. In 2019, the GDP per capita of Taiwan by International Monetary Fund was USD 24,827, which is between Malaysia (USD 11,136) and the Republic of Korea (USD 31,430). (1) GDP: Gross Domestic Product.

Country/region	HDI 2019 (2)	Total number of abstracts
	**Medium HDI (0.550-0.700)**	
**Cambodia**	0.581	4
**Myanmar**	0.584	11
**Bangladesh**	0.614	13
**India**	0.647	107
**Vietnam**	0.693	29
	**High HDI (0.700-0.799)**	
**Indonesia**	0.707	23
**Moldova**	0.711	1
**The Philippines**	0.712	15
**Mainland China**	0.758	115
**Thailand**	0.765	20
**Iran**	0.797	10
	**Very high HDI (0.800-1.000)**	
**Malaysia**	0.804	22
**Taiwan**	Nil*	8
**Republic of Korea**	0.906	7
**Japan**	0.915	12
**UK**	0.920	1
**USA**	0.920	2
**Singapore**	0.935	11
**Hong Kong**	0.939	16
**Total**	/	427

Supplementary Table 2. Faculty members of APSID Schools. APSID: Asia Pacific Society for Immunodeficiencies.

Country/region	Name
	**2015 Hanoi School**
**Hong Kong**	Yu-Lung Lau
**Japan**	Shigeaki Nonoyama
**Switzerland**	Reinhard Seger
**USA**	Roger Hideo Kobayashi
**USA**	Hans D Ochs
	**2016 Hong Kong School**
**Hong Kong**	Yu-Lung Lau
**Hong Kong**	Pamela Pui-Wah Lee
**India**	Surjit Singh
**Israel**	Amos Etzioni
**Japan**	Hirokazu Kanegane
**The Philippines**	Fatima Johanna Santos-Ocampo
**Singapore**	Bee-Wah Lee
**Singapore**	Woei-Kang Liew
**Switzerland**	Reinhard Seger
**Taiwan**	Hsin-Hui Yu
**Thailand**	Narissara Suratannon
**UK**	Andrew Cant
**USA**	Luigi D Notarangelo
**USA**	Hans D Ochs
	**2016 Kuala Lumpur School**
**Hong Kong**	Yu-Lung Lau
**Hong Kong**	Pamela Pui-Wah Lee
**Iran**	Nima Rezaei
**Italy**	Eleonora Gambineri

**Malaysia**	Adli Ali
**Singapore**	Woei-Kang Liew
**USA**	Bob Geng
**USA**	Hans D Ochs
**2017 Chongqing School**	
**Mainland China**	Jing Chen
**Mainland China**	Huawei Mao
**Mainland China**	Xiangfeng Tang
**Mainland China**	Jie Yu
**Mainland China**	Xiaodong Zhao
**Hong Kong**	Yu-Lung Lau
**Hong Kong**	Pamela Pui-Wah Lee
**India**	Surjit Singh
**Japan**	Kohsuke Imai
**Sweden**	Lennart Hammarstrom
**USA**	Hans D Ochs
**2017 Hong Kong School**	
**Australia**	Melanie Wong
**Australia**	Theresa Cole
**Mainland China**	Huawei Mao
**Hong Kong**	Yu-Lung Lau
**Hong Kong**	Pamela Pui-Wah Lee
**Japan**	Tomohiro Morio
**India**	Surjit Singh
**Japan**	Kohsuke Imai
**Singapore**	Woei-Kang Liew
**Switzerland**	Reinhard Seger
**Taiwan**	Wen-I Lee
**USA**	Hans D Ochs
**USA**	Kathleen Sullivan

**2018 Bangkok School**	
**Hong Kong**	Yu-Lung Lau
**Hong Kong**	Pamela Pui-Wah Lee
**India**	Surjit Singh
**Japan**	Kohsuke Imai
**Malaysia**	Amir Hamzah Abdul Latiff
**Switzerland**	Reinhard Seger
**Thailand**	Narissara Suratannon
**UK**	Andrew Gennery
**USA**	Hans D Ochs
**USA**	Panida Sriaroon
**2018 Chongqing School**	
**Australia**	Matthew Cook
**Australia**	Melanie Wong
**Mainland China**	Xiaodong Zhao
**Hong Kong**	Yu-Lung Lau
**Hong Kong**	Pamela Pui-Wah Lee
**India**	Surjit Singh
**Japan**	Hirokazu Kanegane
**Japan**	Kohsuke Imai
**UK**	Andrew Cant
**UK**	Adrian Thrasher
**USA**	Hans D Ochs
**2019 Manado School**	
**Mainland China**	Huawei Mao
**Hong Kong**	Yu-Lung Lau
**Hong Kong**	Pamela Pui-Wah Lee
**Indonesia**	Dina Muktiarti
**Japan**	Hirokazu Kanegane
**Japan**	Hidenori Ohnisi

**Singapore**	Woei-Kang Liew
**Thailand**	Narissara Suratannon
**USA**	Hans D Ochs
**2020 Chandigarh School**	
**Belgium**	Isabelle Meyts
**Mainland China**	Xiaodong Zhao
**Hong Kong**	Yu-Lung Lau
**India**	Amit Rawat
**India**	Biman Saika
**India**	Surjit Singh
**India**	Deepti Suri
**Japan**	Kohsuke Imai
**Malaysia**	Amir Hamzah Abdul Latiff
**The Netherlands**	Taco W Kuijpers
**USA**	Roshini Sarah Abraham
**USA**	Janet Markle
**USA**	Hans D Ochs

References

1. World Economic Outlook Database, October 2019 [Internet]. 2019 [cited 2 April, 2020].
2. United Nations Development Programme. Human Development Report 2019.

End
